# Polarity based characterization of biologically active extracts of *Ajuga bracteosa* Wall. ex Benth. and RP-HPLC analysis

**DOI:** 10.1186/s12906-017-1951-5

**Published:** 2017-09-05

**Authors:** Syeda Saniya Zahra, Madiha Ahmed, Muhammad Qasim, Bilquees Gul, Muhammad Zia, Bushra Mirza, Ihsan-ul Haq

**Affiliations:** 1 Department of Pharmacy, Faculty of Biological Sciences, Quaid-i-Azam University, Islamabad, 45320 Pakistan; 2 Institute of Sustainable Halophytes Utilization, University of Karachi, Karachi, 75270 Pakistan; 3 Department of Biotechnology, Faculty of Biological Sciences, Quaid-i-Azam University, Islamabad, 45320 Pakistan; 4 Department of Biochemistry, Faculty of Biological Sciences, Quaid-i-Azam University, Islamabad, 45320 Pakistan

**Keywords:** *Ajuga Bracteosa*, Protein kinase inhibition, Antiproliferative activity, THP-1 human leukemia cell line, Hep G2 hepatoma cell line, *Leishmania tropica*

## Abstract

**Background:**

The concept of botanical therapeutics has revitalized due to wide importance of plant derived pharmaceuticals. Therefore, the ameliorative characteristics of *Ajuga bracteosa* were studied.

**M**ethods**:**

Total phenolic content, flavonoid content, antioxidant capacity, reducing power and free-radical scavenging activity were determined colorimetrically. Specific polyphenols were quantified by RP-HPLC analysis. Preliminary cytotoxicity was tested using brine shrimp lethality assay while antiproliferative activity against THP-1 and Hep-G2 cell lines was determined by MTT and SRB protocols respectively. Antileishmanial potential was assessed via MTT colorimetric method. To investigate antidiabetic prospect, α-amylase inhibition assay was adopted whereas disc diffusion method was used to detect likely protein kinase inhibitory, antibacterial and antifungal activities.

**Results:**

Among fifteen different extracts, maximum total phenolic content (10.75 ± 0.70 μg GAE/mg DW), total reducing power (23.90 ± 0.70 μg AAE/mg DW) and total antioxidant capacity (11.30 ± 0.80 μg AAE/mg DW) were exhibited by methanol extract with superlative percent extract recovery (17.50 ± 0.80% *w*/w). Chloroform-methanol extract demonstrated maximum flavonoid content (4.10 ± 0.40 μg QE/mg DW) and ethanol extract exhibited greatest radical scavenging activity (IC_50_ 14.40 ± 0.20 μg/ml). RP-HPLC based quantification confirmed polyphenols such as pyrocatechol, gallic acid, resorcinol, catechin, chlorogenic acid, caffeic acid, syringic acid, *p*-coumaric acid, ferulic acid, vanillic acid, coumarin, sinapinic acid, trans-cinnamic acid, rutin, quercetin and kaempferol. The brine shrimp lethality assay ranked 78.60% extracts as cytotoxic (LC_50_ ≤ 250 μg/ml) whereas significant THP-1 inhibition was shown by methanol-acetone extract (IC_50_ 4.70 ± 0.43 μg/ml). The antiproliferative activity against Hep-G2 hepatoma cancer cell line was demonstrated by n-hexane, ethylacetate and methanol-distilled water (IC_50_ 8.65–8.95 μg/ml) extracts. Methanol extract displayed prominent protein kinase inhibitory activity (MIC 12.5 μg/disc) while n-hexane extract revealed remarkable antileishmanial activity (IC_50_ 4.69 ± 0.01 μg/ml). The antidiabetic potential was confirmed by n-hexane extract (44.70 ± 0.30% α-amylase inhibition at 200 μg/ml concentration) while a moderate antibacterial and antifungal activities were unveiled.

**Conclusion:**

The variation in biological spectrum resulted due to use of multiple solvent systems for extraction. We also deduce that the valuable information gathered can be utilized for discovery of anticancer, antileishmanial, antioxidant and antidiabetic bioactive lead candidates.

## Background

The exploitation of natural sources such as plants, animals, and microbes by the humans has been the prime instinctive attempts to combat diseases. Since ancient times a wide array of substances effective against infectious as well as chronic diseases have been obtained from the traditional plants [1]. According to the World Health Organization (WHO) around 65–80% of the developing countries depend mainly on ethnomedicine for their primary health issues because of being easily accessible and economical [2].

In addition to this, an overwhelming issue of disease burden and resistance against already marketed drugs has made high throughput and innovative approaches for drug discovery indispensable [3]. Therefore, the pharmaceutical companies have displayed their active participation in search of lead molecules from higher plants as well as development of standard phytotherapeutic agents with optimum quality, efficacy and safety [2].

One such higher plant with enriched pharmacological properties is *Ajuga bracteosa* Wall. ex Benth. locally known as Kori Booti, (family; Lamiaceae/Labiatae, synonym; *A. remota*). It abounds in the western Himalayas at the altitudes of 1300 m and is widely distributed in subtropical and temperate regions from Kashmir to Bhutan, including Pakistan, Afghanistan, China, and Malaysia. It is a perennial ascending hairy herb, often prostrate with oblanceolate or sub-spathulate leaves and grows up to 5–50 cm tall [4]. The extracts from *A. bracteosa* have been reported to have a wide range of health benefits. Its aerial parts have been used to relieve toothaches and to treat malaria, amenorrhea, rheumatism, palsy, gout and other inflammatory diseases and to act as a diuretic and an antiseptic preparation [5, 6]. The ethanolic extract of *A. bracteosa* demonstrated antiplasmodial activity via in vitro antiplasmodial assay and in vivo schizontocidal activity in infected BALB/c mice [7, 8]. The antiinflammatory effect of the plant was confirmed by using acute and chronic arthritic rat models and mouse ear edema assay [6, 9]. In these studies, ajugarin I, lupulin A, withaferin A, reptoside and 6-deoxyharpagide were found to be responsible for the observed activity. The analgesic effect was reinforced by the tail immersion test and acetic acid induced writhing test in Swiss albino rats [10]. The herbicidal properties of *A. bracteosa* were affirmed by the phytotoxic activity of the n-hexane extract against *Lemna minor* [11]. In addition, the methanol extract of the plant was found to exhibit considerable activity against MCF-7 (human breast adenocarcinoma) and Hep-2 (larynx carcinoma) cell lines [12]. Keeping in view the prodigious biological attributes of *A. bracteosa* its pharmacological spectrum has been further extended in our present study. Fifteen mono and binary solvent systems with escalating polarity have been employed to extensively explore, cytotoxic, protein kinase inhibitory and antileishmanial potential of this plant for the first time.

## Methods

### Solvents and reagents

The analytical grade solvents and reagents were used for the study. The solvents namely n-hexane, ethyl acetate, methanol, ethanol, acetone, chloroform, and dimethylsulfoxide (DMSO) were acquired from Merck (Darmstadt, Germany). The reagents such as 2, 2-diphenyl, 1-picrylhydrazyl (DPPH), Folin–Ciocalteu reagent, sodium hydroxide, aluminum chloride, ferrous chloride, ammonium molybdate, dipotassium hydrogen phosphate, potassium dihydrogen phosphate, sulfuric acid, trichloroacetic acid (TCA), doxorubicin, standard antibiotics (cefixime, roxithromycin, clotrimazole), trypton soy broth (TSB), α-amylase enzyme, acarbose, gallic acid, quercetin, and ascorbic acid were bought from Sigma–Aldrich (Steinheim, Germany). Medium ISP4 was prepared in lab while Sabouraud dextrose agar (SDA) was purchased from Oxoid, England and Tween-20 was bought from Merck-Schuchardt, USA.

### Extraction

In September 2013, *Ajuga bracteosa* plant was collected from the premises of Quaid-i-Azam University. The collected plant was identified by Prof. Dr. Rizwana Aleem Qureshi, department of plant sciences, faculty of biological sciences, Quaid-i-Azam University Islamabad, Pakistan. The shade dried voucher specimen was archived in the Herbarium of medicinal plants, Quaid-i-Azam University Islamabad under herbarium number PHM-496.

After thorough washing under running water to remove intercalating sand particles and debris, the plant material was shade dried at ambient temperature for three weeks. The dried plant material was then finely ground using electric knife mill and subsequently stored in air-tight containers. For extraction, the accurately weighed powder (40 g) was macerated for the period of 24 h with 400 ml solvent in 1000 ml Erlenmeyer flask. The process of maceration was facilitated by ultrasonication in the ultrasonic bath at room temperature for 30 min. Single and binary solvent systems (ratio, 1:1) including n-hexane (Nh), n-hexane-ethyl acetate (Nh-Ea), n-hexane-ethanol (Nh-E), chloroform (C), ethyl acetate (Ea), chloroform-methanol (C-M), ethyl acetate-methanol (Ea-M), acetone (A), methanol (M), acetone-methanol (A-M), acetone-ethanol (A-E), ethanol (E), acetone-distilled water (A-Dw), methanol-distilled water (M-Dw) and distilled water (Dw) were utilized for extraction. A muslin cloth was used to strain the marc from the menstruum followed by filtration through Whatmann No. 1 filter paper. The same procedure was repeated twice and the extracts thus produced were combined and concentrated by evaporation on vacuum in rotary evaporator (Buchi, Switzerland) and further dried in vacuum oven (Yamato, Japan) at 45 °C to obtain final crude extract. The yield of individual extracts of *A. bracteosa* in different solvent systems (mono and binary) was represented by percent extract recovery. The formula to calculate the percent recovery of crude extract is given as follows;

$$ \%\mathrm{Extract}\  \mathrm{recovery}\ \left(\%\mathrm{w}/\mathrm{w}\right)=\left(\mathrm{A}/\mathrm{B}\right)\times 100 $$

A = weight of the dried extract.

B = weight of powdered plant material.

### Preparation of extract solutions

The stock solutions of concentrations 20 mg/ml and 4 mg/ml were prepared in DMSO. The accurately weighed amounts were added to the properly labelled Eppendorf tubes followed by the addition of solvent. Then the samples were ultrasonicated to obtain the clear stock solutions.

### Biological evaluation

#### Phytochemical analysis

##### Determination of total phenolic content (TPC)

The method of estimation of TPC by using Folin-Ciocalteu reagent was adopted with slight modifications, as described previously [13]*.* From each test sample an aliquot of 20 μl (4 mg/ml DMSO) was poured in the designated well of 96 μ well plate followed by addition of 90 μl of Folin-Ciocalteu reagent. Then, 90 μl of sodium carbonate was added to the reaction mixture after an incubation of 5 min. Each reaction mixture was read at 630 nm using microplate reader (Biotech USA, microplate reader Elx 800). Gallic acid was used as a positive control. A calibration curve (y = 0.0163x - 0.0177, R^2^ = 0.9693) was developed by making two fold serial dilutions of gallic acid (6.25–50 μg/ml). Analysis was run in triplicate and the results were presented as μg gallic acid equivalent per mg dry weight (μg GAE/mg DW).

##### Determination of total flavonoid content (TFC)

As reported previously, aluminium chloride colorimetric method was adopted with slight changes to determine total flavonoid content [13]. Each crude extract, 20 μl from 4 mg/ml DMSO stock solution, was poured in 96 well microplate. Then, 10% *w*/*v* aluminum chloride (10 μl) and 1.0 M potassium acetate (10 μl) was added into each well proceeded by 160 μl of distilled water. Subsequently, the reaction mixture was incubated for 30 min at ambient temperature. After incubation, absorbance was recorded at 415 nm using microplate reader. Calibration curve (y = 0.0507x + 0.0179, R^2^ = 0.9983) was plotted by serially diluting the standard, quercetin to the final concentrations 2.5, 5, 10, 20, 40 μg/ml. The assay was performed in triplicate and the values were expressed as μg equivalents of quercetin per mg of plant dry weight (μg QE/mg DW).

##### HPLC-DAD quantitative analysis

The polar extracts Ea, M and Dw were selected for reverse-phase high performance liquid chromatography (RP-HPLC) according to previously described procedure [14]. The methanol (62.5%) and 6 M HCl solutions were used to extract each of the dried extracts (0.5 g). The samples were refluxed for 2 h preceded by purging through nitrogen for few seconds. The extracts were filtered and the volume was adjusted by using methanol to 100 ml and re-filtered through 0.45 μm membrane filter (Millex-HV) before injecting into HPLC. The components of the device consist of column oven (CTO-20A), auto-sampler (SIL-20A), and diode array detector (SPD-M20A) along with HPLC system (Shimadzu LC-20AT). The analytical column Nucleosil C18, 5 μm 100 A° (250 × 4.60 mm, Phenomenex) coupled with a guard column (KJO-4282, Phenomenex) was used. Mobile phase was prepared the ratio of 1% acetic acid solution and 70% methanol. Gradient program was used with a flow rate of 0.8 ml/min [15]. The retention time and chromatographic peaks of UV-Vis spectra of phenolic compounds were obtained and compared with those of authentic reference standards at 280 nm.

### Biological evaluation

#### Antioxidant assays

##### Free radical scavenging assay (FRSA)

The crude extracts were screened for antioxidant activity by using 2, 2-diphenyl 1-picrylhydrazyl (DPPH) which is a stable free radical [16]. An aliquot of 20 μl from test sample (4 mg/ml DMSO) was mixed with 180 μl of DPPH solution (9.20 mg/100 ml methanol) to attain the final concentrations of 7.40, 22.22, 66.66, and 200 μg/ml in the reaction mixture. After 30 min of incubation at 37 °C, the absorbance was measured at 515 nm using microplate reader. The following formula was used to compute %FRSA:

$$ \%\mathrm{FRSA}=\left(1-{\mathrm{Ab}}_{\mathrm{s}}/{\mathrm{Ab}}_{\mathrm{c}}\right)\times 100 $$

Where Ab_s_ and Ab_c_ are the absorbance of sample and negative control respectively. The assay was performed in triplicate by using ascorbic acid as a positive control. The IC_50_ was calculated for the crude extracts exhibiting significant radical scavenging efficiency that is greater than 50% by two fold serial dilution method.

#### Total antioxidant capacity (TAC)

Total antioxidant capacity of the extracts was determined using phosphomolybdenum based assay. A volume of 100 μl of test extract (4 mg/ml DMSO) was mixed with 900 μl of reagent (0.6 M sulphuric acid, 28 mM sodium phosphate and 4 mM ammonium molybdate). DMSO was used as a blank. Then the reaction mixture was incubated in the water bath for 90 min at 95 °C. Upon cooling, the absorbance of the test and standard solutions was measured at 695 nm via PDA spectrophotometer (8354 Agilent Technologies, Germany). The experiment was performed in triplicate. The standard calibration curve (y = 0.0323x  – 0.25, R^2^ = 0.9784) was developed at the concentrations of ascorbic acid (6.25–100 μg/ml) by making two fold serial dilutions. The antioxidant activity was stated as the number of μg equivalents of ascorbic acid per mg of dry weight (μg AAE/mg DW) [13].

#### Total reducing power (TRP)

Potassium ferricyanide colorimetric assay was used to estimate the reducing power of different solvent extracts [13]. An aliquot of 200 μl of each test extract (4 mg/ml DMSO) was added with 400 μl each of phosphate buffer (0.2 mol/l, pH 6.6) and potassium ferricyanide (1% *w*/v in H_2_O) followed by incubation at 50 °C for 20 min. The mixture was then centrifuged at 3000 rpm at room temperature for 10 min after pouring 400 μl of trichloroacetic acid (10% w/v in H_2_O) to each test extract. Then the supernatant (500 μl) was separated and mixed with 500 μl of distilled water and 100 μl of FeCl_3_ (0.1% w/v in H_2_O). The absorbance was then read at 700 nm.. Ascorbic acid was used as a positive control (4 mg/ml DMSO) and DMSO was used as a blank. The equation for ascorbic acid calibration curve (y = 0.0383x + 0.7484, R^2^ = 0.9967) was obtained by making serial dilutions from 6.25 to 100 μg/ml. The reducing power of each sample was expressed as μg ascorbic acid equivalent per mg dry weight (μg AAE/mg DW). The assay was performed in triplicate.

### Cytotoxicity assays

#### Brine shrimp lethality assay

A 96 well microplate was used to perform the brine shrimp lethality assay according to the previously described protocol with little modifications [16]. In order to hatch, the eggs of the test organism *Artemia salina* (Ocean 90, USA) were incubated at 30–32 °C for 24–48 h in the simulated sea water (38 g/l supplemented with 6 mg/l dried yeast) which was presaturated with oxygen. For this purpose, a specially designed tank with two compartments with the porous wall in between was used. In the bigger compartment, eggs were placed and was covered with aluminium foil and the smaller one was kept under constant illumination with the help of a lamp (the hatched nauplii (shrimp larvae) moved phototropically to the other side through the pores). The Pasteur pipette was then used to harvest the nauplii and transferred to a small beaker (50 ml) separately. From the beaker, the shrimp larvae (10 in number) were transferred to the 96 well microplate containing the samples in serial dilutions and the volume was made up with sea water in such a way that DMSO concentration remains less than 1%. The extracts were tested at three graded concentrations i.e. 200, 100, 50 and 25 μg/ml. The solution of doxorubicin (4 mg/ml DMSO) and DMSO served as positive and negative controls respectively. After the period of 24 h, the degree of lethality induced by each extract was quantified by taking into account the number of surviving larvae. The median lethal concentration (LC_50_) of the test samples with ≥50% mortality was calculated using table curve 2D v5.01 software. The whole experiment was run in triplicate analysis.

#### Cytotoxicity against THP-1 human leukemia cell line

The human leukemia (THP-1) cell line (ATCC # TIB-202) was used to assess the in vitro cytotoxicity of the test samples by adopting standard MTT protocol as described previously with slight variations [16]. For this purpose, complete growth medium [RPMI-1640 medium buffered with 2.2 g/l NaHCO_3_ and supplemented with 10% HIFBS (heat inactivated fetal bovine serum); pH 7.4] was used to culture leukemia cells at 37 °C in a humidified carbon dioxide (5%) incubator (Panasonic, Japan MCO-18 AC-PE). To each well of 96 well microplate about 190 μl of THP-1 cells (seeding density 5 × 10^5^ cells per ml) were transferred followed by addition of 10 μl of sample containing not more than 1% DMSO in PBS. Samples were run thrice at the final concentration of 20 μg/ml. The reaction plate was incubated for 72 h at 37 °C in a humidified CO_2_ (5%) incubator. The 5-florouracil and vincristine (both with the concentrations of 4 mg/ml prepared in DMSO) served as positive controls while 1% DMSO in PBS acted as negative control. Afterwards, 20 μl of pre-filter sterilized MTT solution (4 mg/ml in distilled H2O) was added and plates were again incubated at 37 °C for 4 h in humidified CO_2_ (5%) incubator. After incubation supernatant was removed carefully without disturbing colored formazan sediments. To dissolve the formazan sediments 100 μl of DMSO was added in each well, the plate was kept aside for 1 h to ensure full dissolution and the absorbance was measured at 540 nm using microplate reader. Samples showing more than 50% inhibition at 20 μg/ml concentration were further analyzed at lower concentrations i.e. 10, 5, 2.5 and 1.25 μg/ml and the assay was performed in triplicate. IC_50_ was calculated by using table curve 2D v5.01 software.

#### Cytotoxicity against Hep G2 cell line

Cytotoxic potential of the extracts towards Hep G2 cell line (RBRC-RCB1648) was determined by using SRB colorimetric assay as described previously [17]. Dulbecco’s Modified Eagle Medium (DMEM) supplemented with 10% FBS, 100 IU/ml penicillin G sodium, 100 μg/ml streptomycin sulphate and 0.25 μg/ml amphotericin B was used to grow the Hep G2 cells. The plate was then incubated for 72 h in humidified atmosphere enriched with 5% CO_2_ at 37 °C so that a confluence of approximately 70–80% was obtained. The old medium was replaced with fresh medium and the cells were incubated for another 24 h after which they were trypsonised and diluted to get an assay density of 1 × 10^5^ cells/ml. To the 96 well plate, an aliquot of 20 μl from each test sample (containing 1% DMSO in PBS) was transferred followed by addition of 180 μl from above culture to provide the final concentration 20 μg/ml. Doxorubicin (20–0.08 μg/ml) in PBS was used as a positive control while 1% *v*/v DMSO in PBS was used as a negative control. The culture plate was then incubated at 37 °C for 72 h in CO_2_ incubator proceeded by the addition of cold 20% *w*/*v* TCA solution (50 μl) and washing the cells subsequently with tap water (4 times). Then the plate was air dried and stained with 50 μl of 0.057% w/v SRB in 1% w/v acetic acid for 30 min at room temperature. Wells were then washed 4 times with 1% v/v acetic acid and the plates were dried overnight. The 200 μl of 10 mM Tris base (pH 10) was used to solubilize the bound dye and kept for 1 h at room temperature. The optical density was read by a microplate reader (Biotech USA, microplate reader Elx 800) at 515 nm and percent survival was calculated. A zero-day control was performed by adding an equivalent number of cells to sixteen wells, incubating at 37 °C for 1 h and processing as described above. Percent inhibition was calculated using the formula:

$$ \%\mathrm{Inhibition}=100\hbox{--} \left[\left({\mathrm{OD}}_{\mathrm{cells}+\mathrm{tested}\  \mathrm{samples}}-{\mathrm{OD}}_{\mathrm{day}\ 0}\right)/\left({\mathrm{OD}}_{\mathrm{cells}+1\%\mathrm{DMSO}}-{\mathrm{OD}}_{\mathrm{day}0}\right)\ \mathrm{x}\ 100\right] $$

#### Protein kinase inhibition assay

In order to detect the protein kinase inhibitory potential of the test samples prepared from *A. bracteosa*, the strain of *Streptomyces* 85E was utilized [18]. The *Streptomyces* 85E strain was refreshed in the tryptone soya broth medium at 30 °C for 96 h. The bacterial lawn was prepared by spreading spores of refreshed culture onto the sterile plates containing minimal ISP4 medium. The sterile 6 mm filter paper discs were used to load 100 μg extract per disc from the stock solution of 20 mg/ml DMSO. The impregnated paper discs thus produced were then placed on the plates seeded with *Streptomyces* 85E. Surfactin and DMSO infused discs were used as positive and negative controls respectively. The plates were then incubated at 30 °C for 72 h and the results were interpreted as bald and clear zones of inhibition around samples and controls infused discs. The assay was run in triplicate.

### Antimicrobial assays

#### Antileishmanial assay

The extracts were screened to assess antileishmanial potential via MTT colorimetric assay as described previously [19]. The *Leishmania tropica* kwh23 was cultured in Medium-199 supplemented with 10% fetal bovine serum (FBS), 100 IU/ml penicillin G and 100 μg/ml streptomycin sulphate and incubated at 24 °C for 6–7 days. The wells containing almost 5 × 10^6^ promastigotes (180 μl) each were used to test the extracts (20 μl) at the final concentration of 100 μg/ml. Amphotericin-B (0.33–0.004 μg/ml) was maintained as a positive control while 1% DMSO in PBS was used as negative control. This was followed by incubation at 24 °C for 72 h. Then, 20 μl of pre-filter sterilized MTT solution (4 mg/ml in distilled H_2_O) was added and plates were again incubated at 24 °C for 4 h. After incubation supernatant was removed carefully without disturbing colored formazan sediments. To dissolve the formazan sediments 100 μl of DMSO was added in each well, the plate was kept aside for 1 h to ensure full dissolution and the absorbance was measured at 540 nm using microplate reader. Samples showing more than 50% cell mortality at 100 μg/ml concentration were further analyzed at lower concentrations i.e. 33.3, 11.1, 3.7 and 1.23 μg/ml. IC_50_ was calculated by using table curve 2D v5.01 software.

#### Antibacterial assay

To evaluate the antibacterial potential of the test samples of *A. bracteosa*, disc diffusion method was used [13]. The refreshed bacterial cultures of *Staphylococcus aureus* (ATCC-6538), *Bacillus subtilis* (ATCC-6633), *Escherichia coli* (ATCC*-*25922), *Klebsiella pneumoniae* (ATCC-1705) and *Pseudomonas aeruginosa* (ATCC-15442) with pre-adjusted turbidity were used to make lawns on nutrient agar plates. Each test extract (5 μl from 20 mg/ml DMSO), standard (cefexime and roxithromycin, 5 μl from 4 mg/ml DMSO) and negative control (DMSO, 5 μl) was loaded on sterile paper discs and placed on the seeded plates. The average diameter of the zone of inhibition of the sample, standards and control was measured after an incubation period of 24 h at 37 °C. An inhibition zone ≥10 mm in diameter was considered active for the tested samples and further screening was done by three fold microbroth dilution method to determine minimum inhibitory concentration (MIC) [20]. The stock solution (40 mg/ml) of each active sample was used to prepare the master plate of concentration 8 mg/ml in sterile Mueller Hinton broth (so that DMSO does not exceed the final concentration of 1%). Then the samples of the master plate were serially diluted in 96-well microtiter plate with sterile Mueller Hinton broth to obtain the final concentration ranging from 7.41 to 200 μg/ml. Subsequently, a standardized inoculum (5 × 10^4^ CFU/ml) for each bacterial strain was poured in each well. Microtiter plates were then kept at 37 °C for an overnight incubation. The lowest concentration at which the extract exhibited visible growth inhibition was designated as its MIC. The assay was performed in triplicate.

#### Antifungal assay

Antifungal potential of the test samples of *A. bracteosa* was investigated via agar disc diffusion method [13]. The spores of the fungal strains, namely *Fusarium solani* (FCBP-0291), *Aspergillus fumigatus* **(**FCBP-66), *A. flavus* (FCBP-0064), *A. niger* (FCBP-0198) and *Mucor* species (FCBP-0300) were suspended in 0.02% Tween 20 solution. Then 100 μL of each fungal strain of pre-adjusted turbidity against 0.5 McFarland turbidity standard, was swabbed on Sabouraud dextrose agar plates. Sterile filter paper discs were impregnated with 5 μl of test extract (20 mg/ml DMSO), clotrimazole (standard, 4 mg/ml DMSO) and DMSO (negative control) and were placed on the seeded plates. The average diameter (mm) of the zone of inhibition around the extracts, standard and the negative control discs was measured and recorded after an incubation of 24–48 h at 28 °C. The assay was run in triplicate. Extracts producing an inhibition zone ≥10 mm in diameter were screened to determine minimum inhibitory concentrations (MICs) at lower concentrations ranging from 50 to 3.12 μg/disc by standard disc diffusion method. MIC was calculated as the lowest concentration of the extract around which a visible zone of growth inhibition was formed.

#### α-Amylase inhibition assay

Antidiabetic potential of test extracts was determined by α-amylase inhibition assay following the standard protocol with minor modifications [21]. The reaction mixture containing 15 μl phosphate buffer (pH 6.8), 25 μl α-amylase enzyme (0.14 U/ml), 10 μl sample (4 mg/ml DMSO) and 40 μl (2 mg/ml in potassium phosphate buffer) starch solution was incubated at 50 °C for 30 min in 96 well plate followed by addition of 20 μl of 1 M HCl to stop the reaction. Afterwards 90 μl of iodine reagent (5 mM iodine, 5 mM potassium iodide in phosphate buffer) was added to each well. Blank was prepared by adding DMSO and the phosphate buffer instead of plant extract and enzyme solution respectively whereas negative control was prepared by adding DMSO in place of the test extract. Acarbose (250 μM) was used as positive control. Absorbance of reaction mixture was measured at 540 nm. The activity was expressed as percent α-amylase inhibition and calculated by the following equation:

$$ \%\upalpha -\mathrm{amylase}\  \mathrm{inhibition}=\left(\mathrm{Os}-\mathrm{On}\right)/\left(\mathrm{Ob}-\mathrm{On}\right)\times 100 $$

where *On* = Absorbance of negative control, *Os* = Absorbance of sample and *Ob* = Absorbance of blank well.

#### Statistical analysis

The results obtained from cytotoxic, antidiabetic, antimicrobial and phytochemical assays were expressed as mean ± SD from triplicate analyses and were further analyzed statistically by one-way analysis of variance (ANOVA) by using the statistical package PASW Statistics 18.

## Results and discussion

### Yield of the extract

**Fig. 1:**
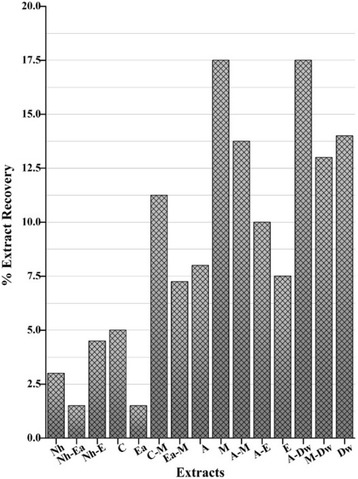
Percent extract recovery of *A. bracteosa* using mono and binary (1:1) solvents for extraction. Nh: n-hexane, Nh-Ea: n-hexane-ethyl acetate, Nh-E: n-hexane-ethanol, C: chloroform, Ea: ethyl acetate, C-M: chloroform-methanol, Ea-M: ethyl acetate-methanol, A: acetone, M: methanol, A-M: acetone- methanol, A-E: acetone-ethanol, E: ethanol, A-Dw: acetone-distilled water, M-Dw: methanol-distilled water, Dw: distilled water

The percent extract yield recovered by employing 15 different solvents and their combinations via the processes of maceration and ultrasonication are depicted in the Fig. 1. By keeping the starting material’s mass and the extraction process constant for all the extracting solvents it was seen that different trends in extract yield were obtained with each single solvent or their combination. The results showed maximum extract yield of 17.50 ± 0.80 and 17.50 ± 0.20% *w*/w in the A-Dw and M solvents respectively while the lowest yields were observed with Nh-Ea and Ea extracts (1.50 ± 0.20 and 1.50 ± 0.40% w/w respectively). Mono and binary solvent systems were used which gave the advantage of wide polarity range for the extractable components from the dried powdered mass of the plant. From the above results it can be postulated that the different solvent systems take up components differently and for maximum extraction a wide range of polarity is used wisely to suit the purpose. It was also seen that overall, nonpolar solvents showed less extraction efficiency than polar ones. Therefore, choice of solvent is a critical factor when it comes to extract recovery [13]. However, this should be kept in mind that the greater extract yield does not mandate the guaranteed biological activity and the medicinal property is dictated by the intrinsic nature of the components which produce the given bioactivity either collectively (confirmed in the fraction or crude form) or individually (confirmed after isolation and purification). The activity may be more pronounced in the low yield solvents or vice versa. This is not determined by the greater yield of the extract. Rather this information is vital for extraction optimization on large scale after a sound activity is observed.

### Phytochemical analysis

#### TPC

**Fig. 2:**
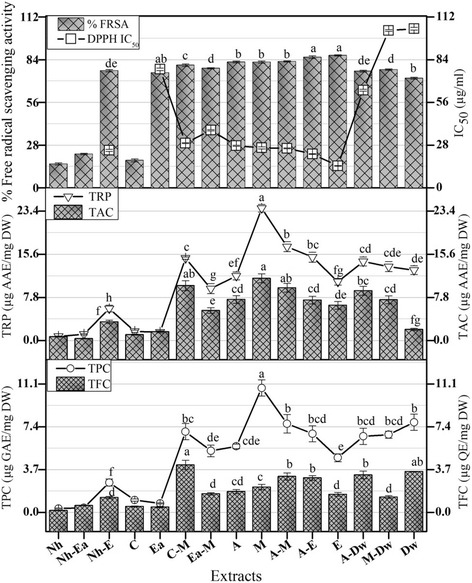
Total phenolic content (μg GAE/mg DW), total flavonoid content (μg QE/mg DW), total reducing power (μg AAE/mg DW), total antioxidant capacity (μg AAE/mg DW) and free radical scavenging activity (%) of *A. bracteosa* in different extracts. IC_50_ of ascorbic acid is found to be 21.8 μg/ml. Values are presented as mean ± Standard deviation (*n* = 3). The columns with different superscript (^a-g^) letters show significantly (*P* < 0.05) different means

The total phenolic content (TPC) of the extracts of *A. bracteosa* prepared in different solvents is given in the Fig. 2. The TPC ranges from the highest value of 10.75 ± 0.70 μg GAE/mg DW produced by M extract to the lowest value of 0.33 ± 0.0 μg GAE/mg DW demonstrated by Nh extract. Overall the total phenolic content is predominately high in extracts prepared in polar solvents or their combinations. The rest of the extracts showed a pattern of decreasing phenolic content in the following order; Dw > A-M > C-M > A-E > M-Dw > A-Dw > A > Ea-M > E > Nh-E > C > Ea > Nh-Ea. Phenolic compounds are ubiquitous, found in virtually all parts of the plant. They are extremely diverse secondary metabolites with a range of pharmacological properties. The studies have shown that phenolics demonstrate antioxidant activity via different mechanisms that is single oxygen quenching, free radical scavenging, metal ion chelation, hydrogen donation, or as substrate for attack by superoxide. Phenolics also inhibit lipid peroxidation by terminating the free radical reactions [22]. In previous studies, *A. iva* was found to contain considerable amount of total phenolic and flavonoid contents [23, 24] suggesting the presence of such substances in *A. bracteosa.* The phenolic compounds obtained from Lamiaceae family give multifaceted activities including neuroprotective, hepatoprotective, antibacterial, antiinflammatory, anticancer, antidiabetic, antiviral, antimycobacterial, antiantherogenic, and antiaflatoxigenic [25].

#### TFC

The total flavonoid content in terms of μg quercetin equivalent per mg dry weight is presented in the Fig. 2. Among all the extracts the highest flavonoid content of 4.10 ± 0.40 μg QE/mg DW was quantified in the C-M extract. While rest of the extracts confirmed the presence of the flavonoid in the following order; Dw > A-Dw > A-M > A-E > M > A > Ea-M > E > M-Dw > Nh-E > Nh-Ea > C > Ea > Nh. Flavonoids are the most widespread and diversified form of phenolics. The flavonoids act mainly via a free radical acceptor mechanism [26]. In order to find whether the antioxidant potential is due to the presence of the flavonoids we found a moderately significant correlation value (R^2^ = 0.6127) which shows that there are other derivatives of phenols responsible for the antioxidant potential along with the flavonoids. According to a study conducted on 14 Japanese *Ajuga* species, flavones such as luteolin, acacetin glycosides, 6-hydroxyluteolin and apigenin and anthocyanins including acylated cyanidin glycosides and acylated delphinidin glycosides were isolated [27]. On the basis of this, it can be concluded that *A. bracteosa* is arich source of flavonoids therefore further studies be carried out to investigate the presence of such flavonoids.

#### RP-HPLC polyphenols profiling

**Fig. 3:**
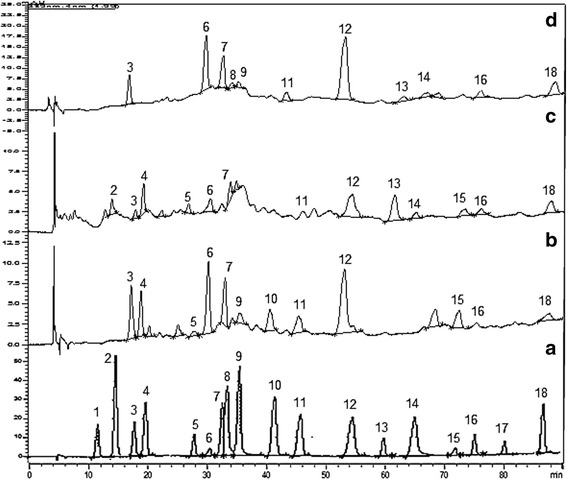
RP-HPLC chromatograms of standard compounds (**a**) and ethyl acetate (**b**), water (**c**), and methanol (**d**) extracts of *Ajuga bracteosa*. Individual peaks showing phenolic compounds i.e. Hydroquinone (1), Pyrocatechol (2), Gallic acid (3), Resorcinol (4), Catechin (5), Chlorogenic acid (6), Caffeic acid (7), Vanillic acid (8), *p*-Coumaric acid (9), Ferulic acid (10), Sinapic acid (11), Coumarin (12), Salicylic acid (13), Trans cinnamic acid (14), Rutin (15), Quercetin (16), Ellagic acid (17) and Kaempferol (18)

The Ea, M and Dw extracts of *A. bracteosa* were screened for the presence of different phenolic compounds via reverse phase HPLC-DAD quantification whose chromatograms are depicted in the Fig. 3 and quantities recorded in Table 1. Among the 18 different polyphenols tested the M extract was found to contain caffeic acid, chlorogenic acid, *p*-coumaric acid, sinapic acid, gallic acid, salicylic acid, kaempferol, quercetin and coumarin compounds while five more compounds; resorcinol, ferulic acid, vanillic acid, rutin, and catechin were detected in Ea extract, and two more phenols (pyrocatechol and trans-cinnamic acid) were found in Dw extract. The Dw extract was found to contain total of 14 different phenolic compounds while 12 and 9 different phenols were detected in Ea and M extracts respectively. Although the M extract contained less number of phenols than the other extracts, but it displayed most prominent free radical scavenging, protein kinase inhibitory and antibacterial bioactivities. On the basis of these results we propose that some polyphenols may produce stronger activity than the others and M extract may have combination of those polyphenols. Moreover, new compounds such as ecdysteroids [28] may be responsible along with these phenols for the said bioactivities.

**Table 1:** Phenolic composition of methanol (M), water (Dw) and ethyl acetate (Ea) extracts of *A. bracteosa*

Classification of phenolic compounds		Retention time	Extracts (mg/g extract)		
			Ea	Dw	M
Phenols	Hydroquinone	10.07	Nd	Nd	Nd
	Pyrocatechol	12.06	Nd	0.071 ± 0.003	Nd
	Resorcinol	17.48	0.239 ± 0.01	0.196 ± 0.02	Nd
Cinnamic acid derivatives	Caffeic acid	27.51	0.224 ± 0.03	0.128 ± 0.01	1.496 ± 0.2
	Chlorogenic acid	25.80	2.032 ± 0.02	0.985 ± 0.04	3.295 ± 0.4
	Ferulic acid	34.87	0.105 ± 0.02	Nd	Nd
	p-Coumaric acid	30.16	0.063 ± 0.002	0.024 ± 0.002	0.121 ± 0.02
	Sinapic acid	50.05	Nd	1.059 ± 0.01	0.860 ± 0.04
	Trans cinnamic acid	54.44	Nd	0.068 ± 0.004	Nd
Benzoic acid derivatives	Gallic acid	15.24	3.495 ± 0.24	0.089 ± 0.005	1.654 ± 0.2
	Salicylic acid	28.21	Nd	Nd	0.124 ± 0.01
	Vanillic acid	38.48	0.127 ± 0.02	0.054 ± 0.003	Nd
Flavonol flavonoid	Kaempferol	73.06	0.103 ± 0.01	0.412 ± 0.04	0.411 ± 0.03
	Quercetin	63.13	0.038 ± 0.015	0.065 ± 0.005	0.220 ± 0.02
flavonol glycoside	Rutin	58.79	0.035 ± 0.003	0.076 ± 0.003	Nd
Flavan-3-ol flavonoid	Catechin	23.61	0.162 ± 0.02	0.203 ± 0.02	Nd
Tannin	Ellagic acid	67.35	Nd	Nd	Nd
Miscellaneous	Coumarin	44.85	0.402 ± 0.03	0.427 ± 0.03	1.689 ± 0.2

Nd Not detected

The polyphenols which are detected in the extracts mainly produce antioxidant effects via radical scavenging either by electron transfer followed by proton transfer, hydrogen atom transfer and sequential proton loss electron transfer [29]. Phenolic acids and flavonoids play important role in lowering risk of most common degenerative and chronic diseases caused by oxidative stress [30, 31].

Polyphenols have documented potential to inhibit cancer cell proliferation, activation of pro-carcinogens, angiogenesis and metastasis and downregulation of drug efflux transporters [32], this can be attributed to sound antioxidant activities of the extracts. Also, a study showed significant relation of polyphenolic content and inhibition of in vivo tumor growth by controlling MAPK/ERK, STAT3, and PI3K/AKT pathways in breast cancer stem cells and reduced lung metastasis [33]. Therefore, the plant may be sought as a potential source of bioactive polyphenolic compounds which would serve as cheap, safe and effective sources of anticancer drugs.

### Biological evaluation

#### Antioxidant assays

##### FRSA

The percent free radical scavenging activity (%FRSA) was measured and the results are shown in the Fig. 2. The highly significant %FRSA from 80 to 87% was produced by the E (86.80 ± 0.40%), A-E (85.60 ± 0.90%), A-M (82.80 ± 0.50%), A (82.50 ± 0.70%), M (82.40 ± 0.70%) and C-M (80.40 ± 0.90%) extracts. The most potent %FRSA was demonstrated by E extract (IC_50_ 14.40 ± 0.20 μg/ml). The %FRSA for the remaining extracts varied in the following sequence; Ea-M > M-Dw > Nh-E > A-Dw > Ea > Dw > Nh-Ea > C > Nh. To find the main components responsible for the free radical scavenging activity of the extracts we developed a correlation with total phenolic content and total flavonoid content (R^2^ = 0.5173 and 0.3757 respectively). The correlation results with TPC values showed that phenols are significantly responsible for the said activity while the correlation results with TFC values made us to infer that there are derivatives other than flavonoid which could result in the radical scavenging activity of the extracts. The antioxidant potential of *A. bracteosa* was previously studied with Nh extract only where it exhibited moderate FRSA activity [11] whereas further studies with other solvents were not reported. On the basis of the present results it is concluded that it has produced good scavenging activity more concentrated in polar extracts than nonpolar ones. Previously, it is shown that plants of the Lamiaceae family exhibit good radical scavenging activity due to the presence of polyphenolic compounds such as romarinic acid, caiffeic acid and ferulic acid [34] which is in agreement with our study.

##### TAC

Total antioxidant activity was analyzed for all the fifteen extracts of *A. bracteosa* and the observations are composed in the Fig. 2. The highest ascorbic acid equivalent was found for the M extract with 11.30 ± 0.80 μg AAE/mg DW. The remaining extracts also showed good activity in the following order; C-M > A-M > A-Dw > M-Dw > A > A-E > E > Ea-M > Dw > Ea > C > Nh > Nh-E > Nh-Ea. Natural antioxidants present in the plant enhance the resistance towards oxidative damages thus producing positive impact on human health [35]. We found a significant correlation between the total phenolic content and the total antioxidant capacity (R^2^ = 0.7185) which explains that the substances producing the antioxidant effect are largely the phenolic compounds. Whereas the correlation between total flavonoid content and total antioxidant capacity (R^2^ = 0.4588) suggests that there may be classes of phenolics responsible for the said activity.

##### TRP

Total reducing power of the extracts was determined and the results were shown in the Fig. 2. The M extract was shown the highest reducing power with 23.90 ± 0.70 μg AAE/mg DW. The other extracts produced reducing power in the following order of activity; A-M > A-E > C-M > A-Dw **>** M-Dw > Dw > A > E > Ea-M > Nh-E > C > Ea > Nh-Ea > Nh. It is evident from the above relationship that the polar extracts have more activity than the nonpolar ones. The control of ‘redox’ status using the plants having antioxidant potential serve great importance as far as balancing the oxidant–antioxidant levels is concerned. Plants produce certain bioactive molecules which render them extremely effective antioxidants due to strong H-donating power. Those molecules may be volatile oils (eugenol, thymol, carvacrol, and menthol), phenolic diterpenes (carnosol, rosmanol, carnosic acid), flavonoids (quercetin, naringenin, catechin, and kaempferol), and phenolic acids (protocatechuic, gallic, caffeic, and rosmadial, and rosmarinic acids) [36]. We found a strong correlation between the total reducing power activity and the total phenolic content (R^2 =^ 0.9676) which shows that in *A. bracteosa* phenols are causing most of the activity. We also found orrelation of the reducing power with the total flavonoid content (R^2^ = 0.5809) which depicts that the said activity is not the sole result of flavonoids; other secondary metabolites are also involved.

### Cytotoxicity assessment

#### Brine shrimp lethality assay

The results of the brine shrimp lethality assay are presented in the Table 2. Most of the extracts were found to be cytotoxic with an LC_50_ ≤ 100 μg/ml. These included the Nh, C, E, A-Dw and M-Dw extracts. It is found that the %mortality crosses the value of 90% at two extremes of polarity range of the extraction solvents. The Nh-Ea, Nh-E, Ea-M, A, M, A-M, and A-E extracts were termed as cytotoxic with the LC_50_ ≤ 250 μg/ml whereas the %mortality of the remaining extracts ranged from 30 to 45% in the following order; C-M > Ea > Dw. Among all the extracts, moderately polar ones such as E, A-Dw and M-Dw were found to be most potent with the LC_50_ of 46.72 ± 0.18, 51.63 ± 1.18 and 63.52 ± 1.70 μg/ml respectively. The positive control used was doxorubicin which exhibited the LC_50_ = 5.93 ± 0.11 μg/ml while DMSO was used as a negative control which exhibited no cytotoxicity. The brine shrimp assay has proved to be a reliable screen for preliminary determination of cytotoxicity of natural products [37]. *A. bracteosa* has shown a promising cytotoxic potential. As far as the effect of polarity is concerned, more potent activity is found to be in the polar extracts as compared to the nonpolar ones. However, the activities of the nonpolar extracts are also moderately significant and can be sought further for a useful source of anticancer lead compounds.

**Table 2:** Cytotoxicity and protein kinase inhibition of different solvent extracts of *A. bracteosa*

Extracts	Brine shrimp cytotoxicity		THP-1 cytotoxicity		Hep G2 cytotoxicity		Protein kinase inhibition		
	% mortality	LC_50_ (μg/ml)	% inhibition	IC_50_(μg/ml)	% inhibition	IC_50_ (μg/ml)	Diameter (mm) at 100 μg/disc		MIC (μg/disc)
	200 μg/ml		20 μg/ml		20 μg/ml		Clear zone	Bald zone	
Nh	63.33 ± 5.77^bc^	91.40 ± 0.23	31.5 ± 1.5	> 20	61.14 ± 0.70^c^	8.95 ± 0.20	10.00 ± 1.0	16.00 ± 1.0^e^	12.5
Nh-Ea	73.33 ± 5.77^b^	112.37 ± 1.20	45.57 ± 1.00^c^	> 20	38.43 ± 0.78	> 20	6.67 ± 0.58	9.67 ± 0.58	100
Nh-E	53.33 ± 5.77^cd^	153.33 ± 1.12	35.87 ± 0.85	> 20	57.37 ± 0.67^d^	19.10 ± 0.50	6.67 ± 0.58	15.00 ± 1.0^e^	25
C	93.33 ± 5.77^a^	85.40 ± 0.26	26.67 ± 0.91	> 20	60.50 ± 0.50^c^	9.50 ± 0.28	6.67 ± 0.58	10.67 ± 0.58^fg^	50
Ea	43.33 ± 5.77	> 200	14.17 ± 1.35	> 20	61.53 ± 0.35^c^	8.63 ± 0.30	6.67 ± 0.58	9.33 ± 0.58	100
C-M	53.33 ± 5.77^cd^	> 200	18.27 ± 2.91	> 20	20.60 ± 0.30	> 20	9.00 ± 1.0	12.00 ± 1.0^f^	100
Ea-M	63.33 ± 5.77^bc^	137.37 ± 0.55	19.63 ± 2.87	> 20	31.45 ± 0.15	> 20	9.00 ± 1.0	30.00 ± 1.0^b^	12.5
A	63.33 ± 5.77^bc^	123.83 ± 0.95	33.13 ± 1.70	> 20	54.71 ± 0.25^e^	19.80 ± 0.20	–	12.00 ± 1.0^f^	100
M	93.33 ± 5.77^a^	110 ± 0.75	45.37 ± 1.56^c^	> 20	56.63 ± 0.20^d^	19.76 ± 0.20	28 ± 1.0	40.00 ± 1.0^a^	12.5
A-M	63.33 ± 5.77^bc^	123.5 ± 0.92	77.63 ± 1.33^b^	4.9 ± 0.20	25.90 ± 0.19	> 20	15.00 ± 1.0	25.0 ± 1.0^c^	50
A-E	93.33 ± 5.77^a^	123.4 ± 0.89	48.27 ± 3.00^c^	> 20	49.80 ± 0.67	> 20	6.67 ± 0.58	12.0 ± 1.0^f^	100
E	96.67 ± 5.77^a^	46.91 ± 0.19	20.8 ± 1.90	> 20	37.40 ± 0.30	> 20	6.33 ± 0.58	7.67 ± 0.58	–
A-Dw	93.33 ± 5.77^a^	52.63 ± 1.11	47.43 ± 2.16^c^	> 20	29.60 ± 0.80	> 20	7.00 ± 1.0	15.00 ± 1.0^e^	50
M-Dw	93.33 ± 5.77^a^	64.93 ± 1.40	30.47 ± 1.95	> 20	63.07 ± 0.40^b^	8.68 ± 0.25	–	12.00 ± 1.0^f^	100
Dw	33.33 ± 5.77	> 200	36.7 ± 1.15	> 20	46.83 ± 0.25	> 20	7.00 ± 1.0	–	–
Doxorubicin	96.67 ± 5.77^a^	5.63 ± 0.25			98.37 ± 0.15^a^	5.30 ± 0.20			
5-Florouracil			99.2 ± 0.92^a^	5.13 ± 0.13					
Vincristine			99.33 ± 0.83^a^	8.24 ± 0.12					
Surfactin								20 ± 1.0^d^	
DMSO	–		–		–			–	

Values are presented as mean ± Standard deviation (*n* = 3). The values with different superscript (^a-g^) letters show significantly (*P* < 0.05) different means. --:No activity

#### Cytotoxicity against THP-I cell line

In 2008, WHO estimated the global incidence of cancer cases as 12.7 million and deaths as 7.6 million. Out of these cases, 56% of cancer cases and 64% of cancer deaths were documented in low and middle income countries. By 2020, the global cancer mortality rate is projected to cross 10 million [38]. Therefore, there is a dire need to explore new candidate molecules with greater efficacy and safety. All the extracts were screened for in vitro cytotoxicity against human leukemia cell line at 20 μg/ml final concentration. The highest cytotoxic activity of 78.10 ± 1.45% was exhibited by A-M extract with an IC_50_ of 4.70 ± 0.43 μg/ml (Table 2). The moderate activity was demonstrated by the Nh-Ea, A-E, A-Dw, and M extracts. The drugs 5-florouracil and vincristine were used as positive controls with an IC_50_ of 5 ± 0.13 and 8.10 ± 0.12 μg/ml respectively. From the results it can be seen that the cytotoxic activity is congregated in the extracts of the moderately polar solvents and their combinations. It can be related to the brine shrimp assay where the cytotoxic activity was stronger in the polar extracts as compared to the nonpolar ones. Previously the methanolic extract of aerial parts of the plant has been reported to exhibit an in vitro activity against human breast adenocarcinoma (MCF-7) and larynx carcinoma (Hep-2) cell lines [12]. This is in accordance with our results which show that moderately polar extracts could be a source of novel compounds. This information should be utilized further through isolation and purification processes to reveal the pharmacologically active constituents of the plant.

#### Cytotoxicity against Hep G2 hepatoma cell line

The results of the *A. bracteosa* extracts tested against Hep G2 cell line are mentioned in the Table 2. The most promising activity was executed by the M-Dw, Ea, Nh and C extracts with the IC_50_ values of 8.65 ± 0.20, 8.78 ± 0.34, 8.95 ± 0.20, and 9.20 ± 0.45 μg/ml respectively followed by Nh-E (IC_50_ = 19.10 ± 0.23 μg/ml), M (IC_50_ = 19.76 ± 0.34 μg/ml) and A (IC_50_ = 19.81 ± 0.20 μg/ml) extracts. In the other extracts, the Hep G2 cell’s inhibition decreased in the following manner; A-E > Dw > Nh-Ea > E > Ea-M > A-Dw > A-M > C-M. The drug doxorubicin was used as positive control with an IC_50_ of 5.1 ± 0.11 μg/ml. From the results it can be deduced that the cytotoxic profile of the *A. bracteosa* extracts mainly accentuates in the nonpolar extracts which assures the presence of active moieties of nonpolar nature. Previously, the aqueous extract of *A. decumbens* is known to display a dose dependent activity against the same cell line in a dose dependent manner which endorses the cytotoxic potential of the plants of the same genus [39]. Contrary to the polar extracts our plant has demonstrated potent activity in the nonpolar extracts and moderately polar extracts. This should be further sought and confirmed through isolation and characterization of the purified compounds from potent extracts.

#### Protein kinase inhibition assay

After a sound in vitro anticancer study, the extracts were further screened for protein kinase inhibitory potential with results stacked in Table 2. The protein kinase inhibitory potential was investigated by using *Streptomyeces* 85E strain. The most potent activity at MIC = 12.5 μg/disc was discovered with M, Ea-M and Nh extracts with the bald zones of 40 ± 1.00, 30 ± 1.00 and 16 ± 1.00 mm at 100 μg/disc respectively. The Nh-E, C, A-M, and A-Dw extracts were found to be moderately active at the MIC of 25–50 μg/disc with the bald zones of 15 ± 1.00, 10.67 ± 0.58, 25 ± 1.00, and 15 ± 1.00 mm at 100 μg/disc respectively. The remaining extracts produced the protein kinase inhibitory activity in the following order; C-M = A = A-E = M-Dw > Nh-Ea > Ea > E. However, clear ZOIs of 28 ± 1.00 and 15 ± 1.00 mm were manifested by M (MIC = 12.5 μg/disc) and A-M (MIC = 50 μg/disc) extracts respectively. The most prominent bald phenotypes were exhibited by the nonpolar to moderately polar extracts. The activity was compared with a known standard, surfactin which showed a bald zone diameter of 20 ± 1.02 mm at 10 μg/disc while DMSO was used as a negative control which showed no toxicity.

The pathogenesis of cancer involves the aggregation of a number of mutations in the genes critical for the cellular growth, differentiation and death. The mutations constitutively elevate kinase activity at serine/threonine residues, commonly found in human cancers [40]. The serine/threonine kinases are a critical factor in carcinogenesis [41]. In the same way, a subfamily of serine/threonine kinases was found to be altered in ovarian carcinomas [42]. The tyrosine kinases regulate the functions of a normal cell and also play a major role in oncogenesis [43]. All these kinases are considered as excellent targets for cancer specific molecules involved in the signaling pathways and to limit nonspecific toxicities observed by conventional therapy [44]. This assay involves the use of *Streptomyces* 85E strain to find the protein kinase inhibitory potential of the plant. The members of *Streptomyces* 85E strain closely resemble with their eukaryotic counterparts and therefore the activity against this strain readily identifies the cytotoxic potential of the samples being tested. Protein kinase inhibitory potential of *A. bracteosa* is reported for the first time which can be further exploited for the drug discovery.

#### Leishmanicidal activity

**Fig. 4:**
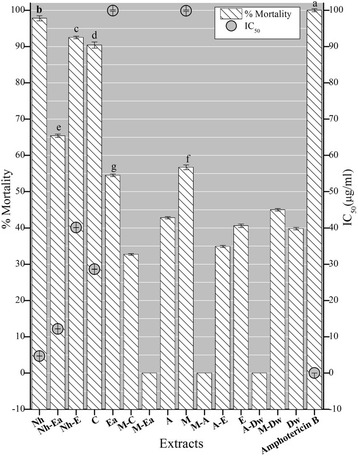
Antileishmanial activity of *A. bracteosa in* different solvents*.* Extracts and the standard are tested at the concentration of 100 and 0.33 μg/ml respectively. Values are presented as mean ± Standard deviation (*n* = 3). The columns with different superscript (^a-g^) letters show significantly (*P* < 0.05) different means

Leishmaniasis is a vector transmitted disease [45] with an annual incidence of 1–1.5 million cases of cutaneous while 500,000 cases of visceral leishmaniasis [46]. According to WHO leishmaniasis is endemic in 88 countries [47] including Pakistan, Afghanistan, India and Sudan [48] with a total of 350 million people at risk [47]. Due to an insufficient impetus towards developing well tolerated, affordable drugs and inappropriate vector (sand fly) control [48] for both prevention and treatment, there is an overall high risk of uncontrolled spread of leishmaniasis [47]. Therefore, 15 *A. bracteosa* extracts were tested against the *Leishmania tropica* and summarized the results in Fig. 4. It was noted that prominent activity was exhibited by Nh, Nh-Ea, C and Nh-E extracts with the IC_50_ of 4.69 ± 0.01, 12.16 ± 0.02, 28.62 ± 0.03 and 40.1 ± 0.02 μg/ml. Moderate activity was exhibited by the Ea and M extracts with the IC_50_ around 100 μg/ml. Further the leishmanicidal activity decreased in the following order: M-Dw > A > E > Dw > A-E > C-M, whereas no activity was seen with Ea-M, A-M and A-Dw extracts. The positive control used was amphotericin B with the IC_50_ 0.01 μg/ml and no activity was observed with DMSO (negative control). The result depicts that the compounds with most potent activity are concentrated in the nonpolar extracts. Previously no such study was reported from this genus. Therefore this information should be further taken up to isolate and characterize pure compounds with potential to develop antileishmanial drugs in future.

#### Antibacterial activity

Limited ability of synthetic compounds in encountering major infections has made the discovery of new molecular lead compounds indispensable [49]. In addition to this, there is an imperative need to look for new antimicrobial moieties against newly emerging infectious diseases [50]. Therefore, traditional plants are being increasingly explored for new leads to develop drugs against microbial infections [51]. The antibacterial potential of the plant was also checked against five different strains of bacteria; the results are shown in the Table 3. Out of all the strains tested, the extracts were found to be more active against *B. subtilis* with M, Ea and Ea-M extracts yielding highest zones of inhibition i.e. 16 ± 0.36, 11 ± 0.23 and 11 ± 0.12 mm at 100 μg/disc respectively. The ZOIs produced by the extracts were comparable to the standard roxithromycin which produced 18 ± 0.43 mm zone of inhibition at 10 μg/disc concentration. The activity was more intensified in non-polar and moderately polar extracts. The negative control used was DMSO which showed no inhibition. While discussing the results it is mentionable that the manifestation of antibacterial action highly depends upon the plant material utilized, methods applied for testing, growth medium composition and the strains used [52]. The extracts were specifically active against *B. subtilis* strain thus qualifying as a valuable source of narrow spectrum antibiotics. Monotherapy with narrow spectrum drugs provides with the advantage of targeted approach which is considered valuable as to avoid the emergence of resistance widely seen in case of broad spectrum antibiotics [53].

**Table 3:** Antibacterial activity of *A. bracteosa* extracts tested against bacterial strains

Extracts	^*^Diameter of growth inhibition zone at 100 μg/disc (mm)									
	*E. coli*	MIC (μg/ml)	*S. aureus*	MIC (μg/ml)	*B. subtilis*	MIC (μg/ml)	*K. pneumoniae*	MIC (μg/ml)	*P. aeruginosa*	MIC (μg/ml)
Nh	7.33 ± 0.58	–	9.33 ± 0.58	–	8.33 ± 0.58	–	7.33 ± 0.58	–	8.33 ± 0.58	–
Nh-Ea	6.67 ± 0.58	–	7.33 ± 0.58	–	6.67 ± 0.58	–	7.33 ± 0.58	–	7.67 ± 0.58	–
Nh-E	7.33 ± 0.58	–	9.33 ± 0.58	–	7.67 ± 0.58	–	7.33 ± 0.58	–	7.33 ± 0.58	–
C	7.67 ± 0.58	–	8.67 ± 0.58	–	9.33 ± 0.58	–	7.33 ± 0.58	–	7.33 ± 0.58	–
Ea	7.00 ± 1.00	–	6.67 ± 0.58	–	10.67 ± 0.58^d^	>100	7.33 ± 0.58	–	8.00 ± 1.00	–
C-M	6.67 ± 0.58	–	6.67 ± 0.58	–	8.33 ± 0.58	–	7.33 ± 0.58	–	8.33 ± 0.58	–
Ea-M	6.67 ± 0.58	–	6.67 ± 0.58	–	10.33 ± 0.58^d^	>100	7.33 ± 0.58	–	9.33 ± 0.58	–
A	6.67 ± 0.58	–	8.33 ± 0.58	–	9.33 ± 0.58	–	7.33 ± 0.58	–	9.33 ± 0.58	–
M	6.33 ± 0.58	–	6.67 ± 0.58	–	16.33 ± 0.58^c^	100	8.33 ± 0.58	–	7.33 ± 0.58	–
A-M	7.33 ± 0.58	–	7.33 ± 0.58	–	8.67 ± 0.58	–	7.67 ± 0.58	–	8.33 ± 0.58	–
A-E	6.67 ± 0.58	–	7.33 ± 0.58	–	9.33 ± 0.58	–	7.33 ± 0.58	–	7.33 ± 0.58	–
E	6.67 ± 0.58	–	7.33 ± 0.58	–	–	–	7.33 ± 0.58	–	8.33 ± 0.58	–
A-Dw	6.67 ± 0.58	–	6.67 ± 0.58	–	8.66 ± 0.58	–	7.33 ± 0.58	–	9.33 ± 0.58	–
M-Dw	6.67 ± 0.58	–	7.33 ± 0.58	–	7.33 ± 0.58	–	9.33 ± 0.58	–	7.33 ± 0.58	–
Dw	6.67 ± 0.58	–	6.33 ± 0.58	–	8.33 ± 0.58	–	7.67 ± 0.58	–	8 ± 1.00	–
Roxithromycin	25.67 ± 0.58^a^	0.334	24.33 ± 0.58^a^	0.334	22.33 ± 0.58^a^	0.334	23.22 ± 0.58^a^	0.334	25.67 ± 0.58	0.334
Cefixime	18.33 ± 0.58^b^	1.11	24.33 ± 0.58^a^	0.334	18.33 ± 0.58^b^	1.11	20.33 ± 0.58^b^	0.334	–	–
DMSO	–		–		–		–		–	–

﻿*Zone of inhibition including the diameter of disc (6 mm)﻿. Values are presented as mean ± Standard deviation (*n* = 3). The values with different superscript (^a-d^) letters show significantly (*P* < 0.05) different means. --: No activity in disc diffusion assay or not active (zone ≥10 mm) for MIC determination

From the review of various studies based on antibacterial properties of the ethnopharmacological sources phenolics were found to be the predominant chemicals responsible for activity against the gram positive bacteria [52]. Further studies also showed the presence of tannins [54] and flavonoids [54, 55]. It can be deduced that this plant is rich in afore mentioned chemicals as confirmed by the phytochemical analysis and it could be a source for natural compounds that can act as a source of new antiinfective agents.

#### Antifungal assay

Extracts of the plant were further evaluated for antifungal activity against five spore forming fungal strains, the results are shown in the Table 4. The most prominent zones of inhibition were found against the three strains that were *F. Solani, A. fumigatus* and *A. flavus* with the values of 14 ± 1.30 (A-Dw extract), 14 ± 0.42 (E extract) and 10 ± 0.34 mm (Dw extract) at 100 μg/disc respectively. The most potent activity (MIC 50 μg/disc) was shown by Nh-E and A-Dw extracts against *F. solani* while the E extract inhibited the *A. fumigatus* at the same value of MIC. The antifungal activity was more pronounced in polar to moderately polar extracts. On the other hand, in case of *F. solani* nonpolar extracts also displayed significant ZOIs in the range of 10–12 mm. Clotrimazole was used as the positive control and displayed the maximum zone of inhibition while DMSO was used as a negative control to rule out any activity given by the solvent. The antifungal activity can be related with the presence of flavonoids, phenolics, tannins and essential oils [56]. The antifungal properties may be attributed to flavonoids as they have the potential to make complexes with extracellular proteins of the fungal cell wall. It also disrupts fungal membranes due to high lipophilicity [57]. Tannins also manifest the same action through similar pathway [58]. Therefore the antifungal activity produced by the plant can be due to the presence of various phenolic compounds whose presence has been confirmed by phytochemical analysis.

**Table 4:** Antifungal activity of *A. bracteosa* extracts

Extracts	^*^Diameter of zone of inhibition (mm)									
	*A. fumigatus*	MIC (μg/disc)	*F. Solani*	MIC (μg/disc)	*A. niger*	MIC (μg/disc)	*A. flavus*	MIC (μg/disc)	*Mucor sp.*	MIC (μg/disc)
Nh	8.67 ± 0.58	–	11.33 ± 0.58^cd^	100	7.33 ± 0.58	–	7.33 ± 0.58	–	7.33 ± 0.58	–
Nh-Ea	6.67 ± 0.58	–	8.33 ± 0.58	–	7.67 ± 0.58	–	6.67 ± 0.58	–	7.33 ± 0.58	–
Nh-E	8.33 ± 0.58	–	12.33 ± 0.58^c^	100	7.33 ± 0.58	–	7.67 ± 0.58	–	6.67 ± 0.58	–
C	7.33 ± 0.58	–	10.33 ± 0.58^d^	100	7.67 ± 0.58	–	7.67 ± 0.58	–	6.33 ± 0.58	–
Ea	7.33 ± 0.58	–	8.33 ± 0.58	–	6.67 ± 0.58	–	8.33 ± 0.58	–	7.33 ± 0.58	–
C-M	7.67 ± 0.58	–	7.33 ± 0.58	–	7.67 ± 0.58	–	7.67 ± 0.58	–	7.33 ± 0.58	–
Ea-M	9.33 ± 0.58	–	7.33 ± 0.58	–	7.33 ± 0.58	–	7.67 ± 0.58	–	7.33 ± 0.58	–
A	7.67 ± 0.58	–	7.67 ± 0.58	–	7.67 ± 0.58	–	7.67 ± 0.58	–	7.33 ± 0.58	–
M	11.33 ± 0.58^c^	100	9.33 ± 0.58	–	8.33 ± 0.58	–	7.67 ± 0.58	–	6.33 ± 0.58	–
A-M	8.33 ± 0.58	–	11.33 ± 0.58^cd^	100	8.33 ± 0.58	–	9.33 ± 0.58	–	6.33 ± 0.58	–
A-E	7.33 ± 0.58	–	7.33 ± 0.58	–	7.67 ± 0.58	–	7.33 ± 0.58	–	7.33 ± 0.58	–
E	13.67 ± 0.58^b^	50	7.33 ± 0.58	–	8.33 ± 0.58	–	7.33 ± 0.58	–	7.33 ± 0.58	–
A-Dw	7.33 ± 0.58	–	14.33 ± 0.58^b^	50	7.33 ± 0.58	–	8.33 ± 0.58	–	7.33 ± 0.58	–
M-Dw	7.33 ± 0.58	–	8.0 ± 0.58	–	7.67 ± 0.58	–	6.67 ± 0.58	–	7.33 ± 0.58	–
Dw	8.67 ± 0.58	–	7.33 ± 0.58	–	8.67 ± 0.58	–	10.33 ± 0.58^b^	100	7.33 ± 0.58	–
Clotrimazole	32.33 ± 0.58^a^	5	29.33 ± 0.58^a^	5	31.67 ± 0.58^a^	5	23.67 ± 0.58^a^	10	41.67 ± 0.58^a^	2.5
DMSO	–		–		–		–	–	–	–

*Zone of inhibition including the diameter of disc (6 mm). In each disc, the sample size was 100 μg per disc (5 μl) in disc diffusion assay. --: No activity in disc diffusion assay or not active (zone ≥10 mm) for MIC determination. Values are presented as mean ± Standard deviation (*n* = 3). The values with different superscript (^a-d^) letters show significantly (*P* < 0.05) different means

### Antidiabetic activity

#### α-Amylase inhibition assay

**Fig. 5:**
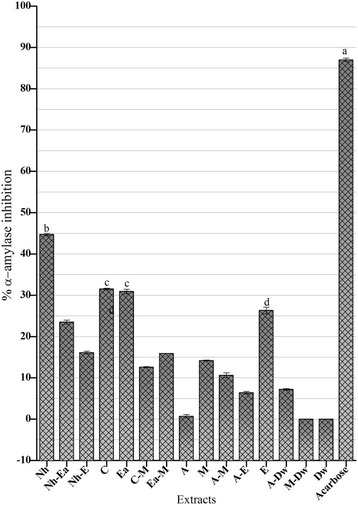
α**-**amylase inhibition assay of *A. bracteosa* extracts. Acarbose is used as a standard with an IC_50_ 33.73 μg/ml. Values are presented as mean ± Standard deviation (*n* = 3). The columns with different superscript (^a-d^) letters show significantly (P < 0.05) different means

Nowadays there is an increasing trend towards using the traditional source of medicines for treatment of the chronic diseases specifically diabetes mellitus; the reason being their safety and low cost [59]. This plant is well known for diabetic control in the folklore medicine and its hypoglycemic effects are documented through a clinical study. This study postulates the antidiabetic potential of the plant which could provide a good control of the symptoms at a low cost and better safety profile. In order to check whether the samples produce antidiabetic effect through alpha amylase inhibition, we tested the extracts by using alpha amylase inhibition assay whose results are compiled in the Fig. 5. The most notable activity was manifested by the Nh extract with the value of 44.70 ± 0.30% α-amylase inhibition. Next to this extract, C and Ea extracts lied with α-amylase inhibition of 31.50 ± 0.20% and 30.90 ± 0.56% respectively. It means that components responsible for the antidiabetic activity are concentrated in the Nh, C and Ea extracts. The activities were compared with the standard drug, acarbose with the demonstrated inhibition of 87 ± 0.43% (IC_50_ = 33.73 μg/ml). In an antidiabetic study involving in vitro and in vivo assays on 5 different *Ajuga* species from Taiwan, flavonoids (which act by reducing oxidative stress) and ecdysterone (which increases insulin sensitivity) were shown to be responsible for the said activity. The activity was concentrated in the nonpolar extracts giving the idea of the nature of the components responsible for the said activity [60].

## Conclusion

The above study featured the importance of using extraction solvents of wide polarity range to unravel the stupendous biological spectrum of *A. bracteosa*. The phytochemical and biological profiling not only endorsed the traditional uses but also brought to light some covert attributes of the subject plant. Our study postulates M extract of *A. bracteosa* as a promising source of antioxidant enriched phytochemicals, with exalted protein kinase inhibitory properties and a sound antibacterial activity against *B. subtilis*. On the other hand, cytotoxic activity against brine shrimps, Hep G2 hepatoma cell line and leishmanial promastigotes was found to be congregated in the C extract. In this regard, further isolation and characterization studies should be conducted to proclaim the bioactive phytochemicals with the said bioactivities.

## Abbreviations

DMSO: Dimethyl sulfoxide
DPPH: 2, 2-Diphenyl-1-picrylhydrazyl
FRSA: Free radical scavenging activity
IC_50_: 50% Inhibitory concentration
LC_50_: Lethal concentration causing 50% mortality
MIC: Minimum inhibitory concentration
TAC: Total antioxidant capacity
TFC: Total flavonoid contents
TPC: Total phenolic contents
TRP: Total reducing power
ZOI: Zone of inhibition
